# Longitudinal dynamics of antibody responses in recovered COVID-19 patients

**DOI:** 10.1038/s41392-021-00559-7

**Published:** 2021-03-31

**Authors:** Meng-Li Cheng, Hui-Ying Liu, Hui Zhao, Guo-Qing Wang, Chao Zhou, Jing Zheng, Xiao-Feng Li, Fan Li, Chang-Qing Bai, Cheng-Feng Qin

**Affiliations:** 1 College of Basic Medical Science, Jilin University, Changchun, China; 2 Department of Virology, State Key Laboratory of Pathogen and Biosecurity, Beijing Institute of Microbiology and Epidemiology, Academy of Military Medical Sciences, Beijing, China; 3 Department of Respiratory and Critical Care Diseases, the Fifth Medical Center, Chinese PLA General Hospital, Beijing, China

**Keywords:** Infectious diseases, Vaccines, Adaptive immunity

**Dear Editor**,

The coronavirus disease 2019 (COVID-19) has resulted in more than 66.5 million cases globally in more than 191 countries with over 1,529,000 mortalities as of 6 December. Currently, the COVID-19 continued to spread around the world, while the duration of antibody lasts and antibody level remained poorly understood, which are primary for safe and effective antiviral treatments and vaccines in the future. In this study, we comprehensively characterized the longitudinal changes of antibodies in recovered patients from 5–7 to 34–42 weeks after symptom onset.

We enrolled 24 COVID-19 recovered patients in this study, including 13 mild and 11 severe patients, respectively. We followed up the discharged patients and collected blood 1–5 times. To better analyze the dynamic responses over time, we divided the samples into 5 time points, including 5–7, 8–10, 11–13, 14–16, and 34–42 weeks after symptom onset, and each group all included mild/severe recovered patients.

We firstly measured the IgG and IgM titers by ELISA. The IgG antibody was decreasing from 5–7 to 34–42 weeks (Fig. 1a), while there was no significant difference among different time points. Obviously, the IgG titers of the severe were higher than that of the mild, and statistically higher during 14–16 and 34–42 weeks (Fig. 1b). In addition, the IgG titers did not decline significantly over time in either mild or severe patients (Fig. 1b). These data suggested the IgG antibody against SARS-CoV-2 could be stable till 34–42 weeks in the recovered patients.

**Fig. 1:**
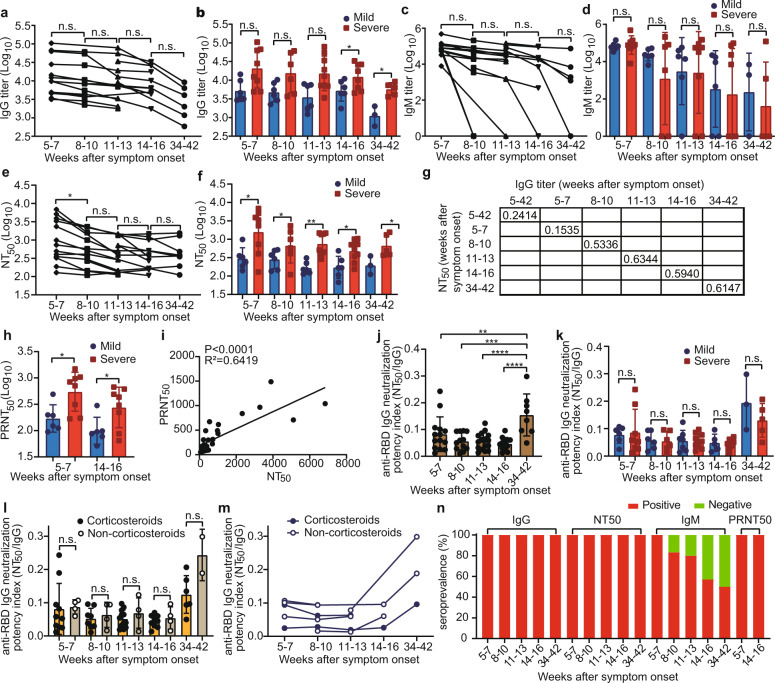
Antibody responses against SARS-CoV-2 spike protein in COVID-19 recovered individuals. IgG (**a**), IgM (**c**), and NT_50_ titers (**e**) against SARS-CoV-2 S-RBD or S protein of the recovered patients from 5–7 to 34–42 weeks (*n* = 17). Comparison of the IgG (**b**), IgM (**d**), and NT_50_ titers (**f**) between mild and severe patients from 5–7 to 34–42 weeks (*n* = 24). **g** Correlation analysis of NT_50_ titers and IgG titers of the recovered patients during 5–42, 5–7, 8–10, 11–13, 14–16, and 34–42 weeks. **h** Comparison of the PRNT_50_ titers between mild and severe patients during 5–7 (*n* = 14) and 14–16 (*n* = 14) weeks. **i** Correlation analysis of NT_50_ titers and PRNT_50_ titers of the recovered patients during 5–16 weeks (*n* = 28). Anti-RBD IgG neutralization potency index (NT_50_/IgG) was calculated (**j**) and compared between mild and severe patients (**k**) from 5–7 to 34–42 weeks (*n* = 24). **l** Comparison of the anti-RBD IgG neutralization potency index (NT_50_/IgG) between the patients who treated with corticosteroids or not from 5–7 to 34–42 weeks (*n* = 24). **m** The longitudinal dynamics of the anti-RBD IgG neutralization potency index (NT_50_/IgG) in mild patients who treated with corticosteroids or not (*n* = 7). **n** Seroprevalence of the IgG, IgM, NT_50_, and PRNT_50_ titers in patients from 5–7 to 34–42 weeks after symptom onset (5–7, *n* = 14; 8–10, *n* = 12; 11–13, *n* = 17; 14–16, *n* = 10; 34–42, *n* = 8). n.s., not significant; *P < 0.05; **P < 0.01; ***P < 0.001; ****P < 0.0001

The IgM antibody against SARS-CoV-2 of the recovered subjects was all positive during 5–7 weeks, while during 8–11 weeks, 16.7% of the patients were negative for the IgM (Fig. 1c, d). And the number of negative samples for IgM increased gradually during the followed weeks (Fig. 1c, d). Compared with IgM during 5–7 weeks, there was no significant difference during 8–10 and 11–13 weeks, while showing significant reduction during 14–16 weeks (Fig. 1c, d).

Neutralizing antibodies are the critical correlates of protective immunity. As shown in Fig. 1e, f, with the extension of time, the NT_50_ titers were in a decreasing line from 5–7 to 11–13 and then maintained stable till 34–42 weeks. During 5–7 weeks, the average NT_50_ titers were at a high level of 1929, while showed a significant drop to 644.6 during 8–10 weeks. Interestingly, the mean NT50 titers of the 3 followed time points maintained at a similar level that slightly lower than that during 8–10 weeks. Of note, the NT_50_ titers of severe patients were significantly higher than that of the mild from 5–7 to 34–42 weeks (Fig. 1f). We further compared the NT_50_ titers between patients who treated with corticosteroids or not, while the NT_50_ titers of the corticosteroids-patients were slightly higher than the non-corticosteroids-patients (Supplementary Fig. S1a). Then we found that the NT_50_ titers of the non-corticosteroids-patients were slightly higher than the corticosteroid patients from the limited number of mild patients (Supplementary Fig. S1b, c). It indicated that the humoral immune responses were more robust in severe patients and corticosteroids may have blunted the neutralization response.^1^

Furthermore, regression analysis was performed on NT_50_ and the corresponding IgG titers (Fig. 1g). The correlation was weak of all the samples from 5 to 42 weeks. Then we analyzed the correlation at each time point separately. During 5–7 weeks, the *R*^2^ was only 0.1535, then increased to 0.5336, 0.6344, 0.5940, and 0.6147 during 8–10, 11–13, 14–16, and 34–42 weeks, respectively, which was representing in an increasing line from 5–7 to 11–13 weeks and maintained stable till 34–42 weeks.

We also conducted a standard plaque reduction neutralization test (PRNT) to confirm the neutralization capacity and calculated the 50% neutralization titers (PRNT_50_). Consistent with NT50 titers, PRNT50 titers of the severe was significantly higher than that of the mild during 5–7 and 14–16 weeks, and still maintained at a high level of 256.8 during 14–16 weeks (Fig. 1h). Moreover, the correlation was very strong between NT_50_ and PRNT_50_ titers (Fig. 1i). A recent study^2^ has indicated that a 1:30 titer measured by PRNT_50_ reduced the risk of getting reinfection by 50%. According to the strong correlation between NT_50_ and PRNT_50_ titers in our study, and the geometric median and arithmetic mean of the ratios of NT_50_ and PRNT_50_ titers are 2.34 and 2.58, respectively, so we suppose that the NT_50_ titer is at least 70.2 that the risk of reinfection can be reduced by 50%.

Next, we calculated the neutralization potency index (NT_50_/IgG) to predict disease severity and survival as previously described.^1^ The neutralization potency index was similar from 5–7 to 14–16 weeks, while was significantly higher during 34–42 weeks (Fig. 1j). Besides a slightly higher in mild patients during 34–42 weeks, there was no difference between mild and severe patients from 5–7 to 14–16 weeks (Fig. 1k). Consistent with NT_50_ titers, the neutralization potency index of non-corticosteroids-patients was similar to corticosteroid patients in all enrolled patients (Fig. 1l), while was slightly higher in mild patients (Fig. 1m, Supplementary Fig. S1). Of note, the neutralization potency index showed an obvious increasing trend during 34–42 weeks.

Finally, we analyzed the positive proportion of IgG, IgM, and neutralizing antibodies in the recovered patients (Fig. 1n). The positive proportion of IgG and neutralizing antibodies were both 100% till 34–42 weeks after symptom onset. The proportion of patients with positive IgM antibody was 100% during 5–7 weeks, then gradually decreased to 50.0% during 34–42 weeks. These results suggested that IgM antibody faded away in the recovered patients over time.

In our study, it is the first time to describe the correlation between IgG and neutralizing antibodies in detail. During 5–7 weeks the *R*^2^ was only 0.1535, which may account for the wave of short-lived, low-affinity antibodies generated by plasmablasts during acute SARS-CoV-2 infection, which then gradually degraded and the germinal center responses that generated high-affinity antibodies can maintain for months to many years.^3^ We find that the strong correlation between IgG and neutralizing antibodies was appeared during 11–13 weeks, while the plasmablasts are no longer present in recovered individuals at ~1 month after symptom onset,^4^ that on one hand it probably because of short-lived, low-affinity antibodies degraded later than plasmablasts disappeared, on the other hand, it possibly account for the time distance between plasmablasts disappeared and the germinal center generated high-affinity antibodies.

The humoral immune responses play a critical role in the clearance of cytopathic viruses and prevention of viral reinfection. Since the outbreak of COVID-19, scientists are committed to exploring the nature and durability of the humoral immune response. Recently, Ripperger et al.^5^ found that neutralizing antibodies were durable at least 2–3 months and Wajnberg et al.^2^ found that more than 90% were detectable for at least 5–7 months after SARS-CoV-2 infection in the mild-to-moderate COVID-19. While in our cohort, the positive proportion of neutralizing antibodies was 100%, and the NT_50_ titers in all patients were >110 till 34–42 weeks after symptom onset. Our results indicated that the high-affinity and efficiency-neutralizing antibodies could last for at least 10 months and supported protective immunity in the recovered patients, and provided insight on the rational development of neutralizing antibodies based on preventive and therapeutic strategies against COVID-19 in the future.

## Supplementary information

Supplementary Materials

## References

[1] Garcia-Beltran WF (2021). COVID-19-neutralizing antibodies predict disease severity and survival. Cell.

[2] Wajnberg A (2020). Robust neutralizing antibodies to SARS-CoV-2 infection persist for months. Science.

[3] Slifka MK, Ahmed R (1996). Long-term antibody production is sustained by antibody-secreting cells in the bone marrow following acute viral infection. Ann. N. Y. Acad. Sci..

[4] Mathew D (2020). Deep immune profiling of COVID-19 patients reveals distinct immunotypes with therapeutic implications. Science.

[5] Ripperger TJ (2020). Orthogonal SARS-CoV-2 serological assays enable surveillance of low-prevalence communities and reveal durable humoral. Immun. Immun..

