# Supplementary material for: Longitudinal dynamics of antibody responses in recovered COVID-19 patients
Source: Signal Transduct Target Ther. 2021 Mar 31;6:137. doi: 10.1038/s41392-021-00559-7 (PMC8009921; doi:10.1038/s41392-021-00559-7)
Supplement: Supplementary file 1 — Supplementary Materials [file 41392_2021_559_MOESM1_ESM.docx]

Supplementary Materials for

Longitudinal dynamics of antibody responses in recovered COVID-19 patients

Meng-Li Cheng, Hui-Ying Liu, Hui Zhao, Guo-Qing Wang, Chao Zhou, Jing Zheng, Xiao-Feng Li, Fan Li, Chang-Qing Bai, Cheng-Feng Qin

Correspondence to: Cheng-Feng Qin (qincf@bmi.ac.cn)

**This PDF file includes:**

**Figures. S1 to S2**

**MATERIALS AND METHODS**

**Supplementary figures and figure legends**


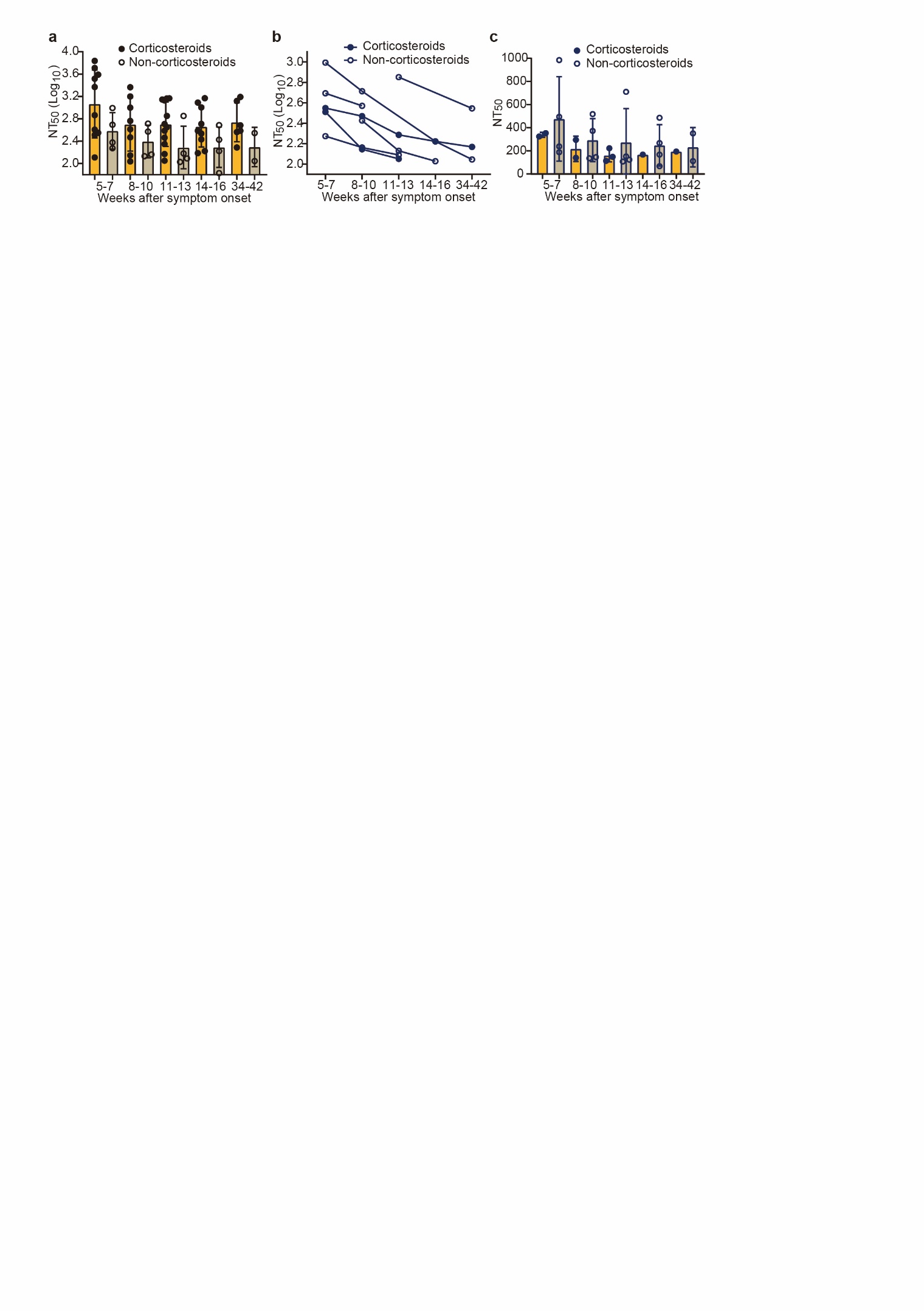


**Supplementary Fig. S1** Comparison of the NT_50_ titer between the corticosteroids and non-corticosteroids in all patients (n=24) (**a**) and in mild patients (n=13) (**c**) from 5-7 to 34-42 weeks (n=24). **b** The longitudinal dynamics of NT_50_ titer in mild patients who treated with corticosteroids or not (n=7).


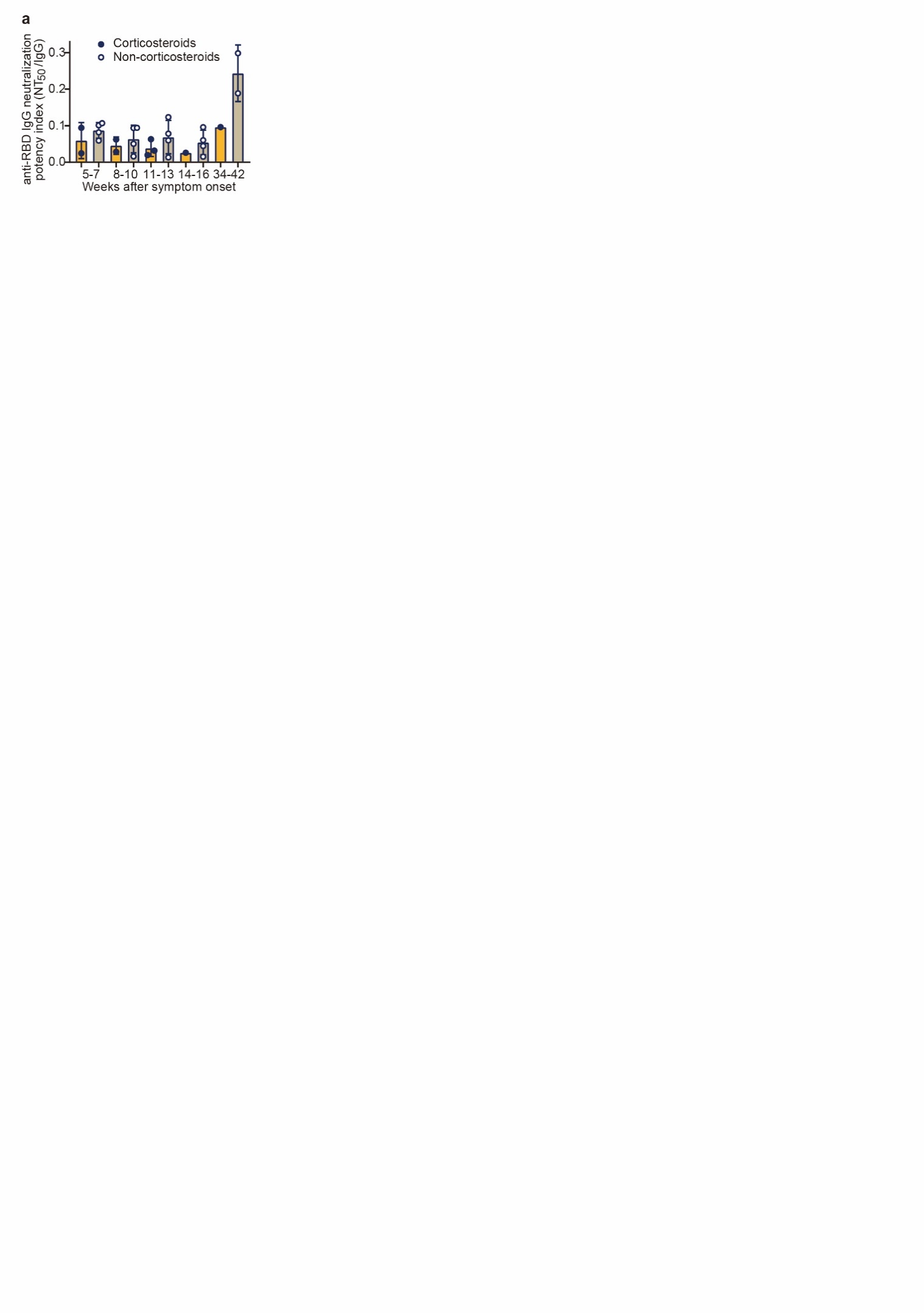


**Supplementary Fig. S2** Comparison of the anti-RBD IgG neutralization potency index (NT_50_/IgG) between corticosteroids and non-corticosteroids in mild patients from 5-7 to 34-42 weeks (n=13).

**MATERIALS AND METHODS**

**Human samples and study design**

Study participants were recruited at the Fifth Medical Center of Chinese PLA General Hospital in China, Beijing. All participants signed an informed consent form before enrollment, and the study was reviewed and approved by the Fifth Medical Center of Chinese PLA General Hospital (2020031D).

COVID-19 case definition and clinical classification based on severity were defined according to the New Coronavirus Pneumonia Prevention and Control Protocol for COVID-19 (6th edition) released by the National Health Commission of China. Further, the 24 enrolled patients were divided into two groups according to clinical classification, that mild and common are into the “mild” group (n=13), severe and critical are into the “severe” group (n=11). To study the dynamics of antibody responses, blood samples were collected successively. The 24 recovered patients were followed up from 5 to 42 weeks after symptom onset, of whom were tested for 1 to 5 times. Among the 24 patients, 17 patients were tested for at least twice, which included 7 in mild group and 10 in severe group.

**Pseudovirus neutralization assay**

The SARS-CoV-2 pseudovirus neutralization assay was conducted as described previously[^1^](#_ENREF_1)^,^[^2^](#_ENREF_2). In brief, 3-fold serial dilution serum (100 μl) from the subjects were incubated with the SARS-COV-2 pseudovirus containing 650 TCID_50_ (50 μl) for 1 hour at 37 °C, then 2×10^4^ Huh7 cells (100 μl) were seeded to each well for 24 hours at 37 °C. After the incubation, 150 μl of the supernatant was removed, 100 μl of luciferase substrate was added to each well and incubated for 2 min in darkness at room temperature. Then luciferase activity was measured using GloMax® 96 Microplate Luminometer (Promega). The 50% nertralization titer (NT_50_) was defined as the serum dilution at which the relative luminescence units (RLUs) were reduced by 50% compared with the virus control wells. Finally, NT_50_ were determined by non-linear regression, i.e. log (inhibitor) vs. normalized response-Variable slope, using GraphPad Prism 7.0 (GraphPad Software, Inc., San Diego, CA, USA).

**PRNT_50_ assay.**

PRNT_50_ assay was performed as described previously[^2^](#_ENREF_2). In brief, Vero cells were seeded in 24-well plates (2x10^5^ cells/well) and incubated for approximately 16 h until 90-100% confluent. Serial 3-fold dilutions of serum were prepared in a 48-well plate and pre-incubated with titerated virus in a 1:1 (vol/vol) ratio to generate a mixture containing ~200 PFU/ml of viruses for 1 h at 37 °C, and DMEM was used as negative control. The virus/serum mixtures were added in the Vero cells and incubated for 1 h at 37 °C. Then the mixtures were removed and added DMEM contain 2% FBS and 1% low-melting point agarose (Promega). 2 days later, the cells were fixed with 4% formaldehyde and stained with 0.2% crystal violet. The PRNT_50_ was calculated by the method of Spearman-Karber.

**References.**

1. Nie, J. *et al.* Establishment and validation of a pseudovirus neutralization assay for SARS-CoV-2. *Emerging microbes & infections* 9, 680-686 (2020).

2. Zhang, N. N. *et al.* A Thermostable mRNA Vaccine against COVID-19. *Cell* 182, 1271-1283 e16 (2020).
